# Disrupted postnatal lung development in heme oxygenase-1 deficient mice

**DOI:** 10.1186/1465-9921-11-142

**Published:** 2010-10-10

**Authors:** Tiangang Zhuang, Monica Zhang, Huayan Zhang, Phyllis A Dennery, Qing S Lin

**Affiliations:** 1 Division of Neonatology, Children's Hospital of Philadelphia, Philadelphia, PA 19104 USA; 2 Department of Pediatrics, Division of Neonatology, University of Pennsylvania School of Medicine, Philadelphia, PA 19104 USA

## Abstract

**Background:**

Heme oxygenase (HO) degrades cellular heme to carbon monoxide, iron and biliverdin. The HO-1 isoform is both inducible and cyto-protective during oxidative stress, inflammation and lung injury. However, little is known about its precise role and function in lung development. We hypothesized that HO-1 is required for mouse postnatal lung alveolar development and that vascular expression of HO-1 is essential and protective during postnatal alveolar development.

**Methods:**

Neonatal lung development in wildtype and HO-1 mutant mice was evaluated by histological and molecular methods. Furthermore, these newborn mice were treated with postnatal dexamethasone (Dex) till postnatal 14 days, and evaluated for lung development.

**Results:**

Compared to wildtype littermates, HO-1 mutant mice exhibited disrupted lung alveolar structure including simplification, disorganization and reduced secondary crest formation. These defects in alveolar development were more pronounced when these mice were challenged with Dex treatment. Expression levels of both vascular endothelial and alveolar epithelial markers were also further decreased in HO-1 mutants after Dex treatment.

**Conclusions:**

These experiments demonstrate that HO-1 is required in normal lung development and that HO-1 disruption and dexamethasone exposure are additive in the disruption of postnatal lung growth. We speculate that HO-1 is involved in postnatal lung development through modulation of pulmonary vascular development.

## Background

Despite the dramatic advances in modern neonatal care for premature infants, bronchopulmonary dysplasia (BPD) remains a major cause for morbidity and mortality in extremely premature infants born at 23-28 weeks of age. The central pathophysiological hallmarks of BPD include arrested alveolar development and impaired pulmonary vascularization, which result in a simplified alveolar structure with reduced surface gas exchange area and compromised pulmonary function. Normal lung development is a complex process, highly coordinated by growth factors, signaling molecules, transcription factors, hormones and antioxidant enzymes to direct cell fate determination, branching morphogenesis, vascularization and alveolarization[1,2]. Disruption of alveolarization correlates directly with decreased lung compliance in pulmonary function tests both in patients with bronchopulmonary dysplasia (BPD) and in rodent models[3,4]. Certain conditions such as hypoxia, hyperoxia, or treatment with corticosteroids inhibit lung alveolarization, whereas treatment with retinoic acid and vitamin D promote alveolar development[5-8]. However, the exact mechanisms regulating alveolar development are not completely understood, as it requires interactions between multiple cell types, each of which responds to a variety of growth factors, hormones, and environmental conditions[9].

Increased oxidative stress contributes significantly to the development of BPD in preterm infants who often require ventilation and oxygen therapy. Heme oxygenase (HO) is an anti-oxidant molecule that catalyzes the degradation of cellular heme to carbon monoxide (CO), free iron, and bilirubin [10,11]. Two isoforms, the inducible HO-1 and the constitutively expressed HO-2, have been identified in a wide range of tissues including the lung [12,13]. In the lung, expression of the inducible HO-1 isoform peaks in the perinatal period, a critical phase for alveolar development, then decreases to adult levels [14,15]. At the cellular level, HO-1 is expressed in multiple lung cell types including alveolar type II epithelial cells, macrophages, vascular smooth muscle and endothelial cells [16-18]. HO-1 gene expression can be dramatically induced by hyperoxia, hypoxia, heavy metals, oxidized LDL and inflammation amongst other injuries [13,19]. Induction of HO-1 has been reported in patients with impaired lung alveolar structure, such as acute respiratory distress syndrome (ARDS), chronic obstructive pulmonary disease (COPD) and cystic fibrosis (CF) [20-22]. In cell and animal models, the induction of HO-1 plays a cyto-protective role in response to oxidative stress, inflammation, and lung injury [23-28]. HO-1 is also involved in vascular development as it facilitates blood vessel formation in tumors, wounds, and experimental models of angiogenesis [19,29].

Mice with a targeted HO-1 mutation show partial penetrance of embryonic lethality, growth retardation and deficiency in iron metabolism [19,30,31]. Embryonic fibroblasts generated from these mice have high free oxygen radical production and display hypersensitivity to cellular toxins, indicating that the lack of HO-1 may make mutants more susceptible to injury and stress [31]. The exact cause of this lethality and growth retardation, however, has not yet been determined. To date, only one human patient with HO-1 deficiency has been reported. This individual displayed severe and persistent endothelial cell damage, which was dramatically enhanced by further oxidative stress [32].

Antenatal and postnatal steroid therapy benefits preterm infants by accelerating lung maturation, reducing lung inflammation and facilitating extubation from the ventilator [33,34]. However, the adverse effects associated with glucocorticoid usage are significant and there also may be detrimental long-term damage to the brain and lung [35,36]. In the lung, dexamethasone can impair lung septation and alveolar formation in early postnatal age. Molecular mechanisms of the hormonal effects and its interactions with other signaling molecules are not well understood yet.

To better understand the mechanism by which HO-1 affects lung development in the neonatal period, we evaluated the lung histology and gene expression of alveolar type II epithelial cell and vascular cell markers in wildtype and HO-1 null littermates. Furthermore, we also compared the effect of postnatal dexamethasone on these parameters between wild type and HO-1 null neonates. Results of these studies suggest that there is abnormal alveolar development and expression of cell specific genes in mice lacking HO-1 and that this disruption of lung development is additive to the effects of postnatal dexamethasone.

## Methods

### Animals and Dexamethasone treatment

Mice were housed at the Stokes Institute Laboratory Animal Facility under pathogen-free conditions on a 12:12 h dark-light cycle with unlimited access to food and water. All protocols were reviewed and approved by the Stokes Institutional Animal Care and Use Committee and in accordance with the Animal Welfare Act and the National Institutes of Health guidelines for the care and use of animals in biomedical research.

Wildtype C57BL/6 mice were purchased from commercial vendors. Within 12 hours of birth, newborn mice were randomly split into two groups and injected subcutaneously each day from postnatal day 3 (P3) to P14 with 20 μl of saline (0.9% NaCl) with or without dexamethasone (Dex, 1 μg/pup/day in saline).

In each experiment with HO-1 knockout mutants, at least two litters of newborn mice from HO-1 +/- breeding were randomly selected for control and Dex treatment. The newborn animals were injected subcutaneously each day from P3 to P14 with 20 μl of saline with or without Dex (0.25 μg/pup/day). Genotypes of the animals were determined by PCR with tail biopsies obtained at time of sacrifice [30].

### Lung tissue collection and histology

Mice were sacrificed at two time points, P10 and P14. Mice were anesthetized by intraperitoneal injection of Ketamine/Xylazine. After the pulmonary artery was perfused with 1X PBS, the right lung was excised and snap-frozen with liquid nitrogen, providing samples for protein and RNA analysis. The left lung was inflated to 20 cm H_2_O pressure and fixed with 4% formaldehyde overnight. Lung tissue was paraffin-embedded and 5- μm thick sections were mounted on glass slides and stained with hematoxylin and eosin (H&E).

### Radial alveolar count (RAC)

Alveolarization was quantified by performing radial alveolar counts (RAC), as described [37,38]. Briefly, respiratory bronchioles were identified as bronchioles lined by epithelium in one part of the wall. A perpendicular line was drawn from the center of the respiratory bronchiole to the distal acinus (either the pleura or the nearest connective tissue septum). A minimum of forty lines for each lung was drawn and the number of septae intersected by each line counted. In addition, at least three sections from several levels within the tissue block were used for each animal.

### Determination of HO protein levels

HO protein levels in the lung were examined for wildtype animals treated with saline or Dex. Whole lung homogenates from the snap-frozen right lungs were subjected to Western blot analysis with primary antibodies (Stressgen, 895 for HO-1 and 897 for HO-2), secondary antibodies and ECL reagents (Amersham Biosciences). Equal loading was verified with Western blot analysis using actin antibodies (SC-7210, Santa Cruz Biotechnology). Protein levels were quantified by densitometric analysis (BioRad Quantity One).

### RNA and Quantitative real-time PCR (qRT-PCR) Analysis

Total RNA was extracted from the snap-frozen lung tissues using Trizol reagent (Invitrogen). 200 ng of total RNA were reverse transcribed with random primers and Superscript III enzyme (Invitrogen). Real-time PCR was performed in 384-well format with ABI Prism SDS 7900 HT (Applied Biosystems) according to manufacturer's instructions. 5% of each reverse transcription reaction was used in real-time PCR with gene specific Taqman assays (Applied Biosystems). These assays are: 18 S (Assay ID: Hs99999901_s1), SP-A (Assay ID: Mm00499170_m1), SP-B (Assay ID: Mm00455681_m1), SP-C (Assay ID: Mm00488144_m1), SP-D (Assay ID: Mm00486060), Flk-1 (Assay ID: Mm01222419_m1), and Tie2 (Assay ID: Mm0001256904_m1). SDS 2.3 program was used to calculate delta Ct values normalized to 18 S. Relative quantification of mRNA expression was determined by the delta delta CT method, and presented as ratio to the wildtype, or control treatment group level.

### Statistical Analysis

Data from three or more independent experiments were collected and analyzed as mean ± SEM. For comparison between treatment groups, the Null hypothesis that there is no difference between treatment means will be tested by a single factor analysis of variance (ANOVA) for multiple groups or unpaired t-test for two groups. The significance of the results was assessed by a paired t test between two groups. A p value <0.05 was considered significant.

## Results

### HO-1 mutants displayed partial embryonic lethality

To generate HO-1 homozygous null mutants (HO1-/-), HO-1 heterozygous animals (HO1+/-) were time-mated. Genotypes of the offspring were determined by PCR using wildtype and HO-1 mutant allele specific primers. Compared to the wildtype littermates, the viable HO-1-/- neonates were smaller in body size and less active. Instead of the expected Mendelian ratio of 25%, we identified only 9.9% of the offspring as homozygous mutants (n = 221), indicating that HO-1 knockout mice display partial embryonic lethality. To determine the critical stage when HO-1-/- embryos were dying, staged embryos were harvested from HO-1+/- breeding pairs. Viable embryos identified by a visible beating heart at dissection were genotyped with the same PCR strategy. At E15.5, we recovered viable HO-1-/- embryos at a similar ratio as wildtype littermates. However, at E18.5, viable HO-1 -/- embryos had decreased to 16.2%, and live P1 pups represented only 11.6% of the offspring (Table 1). These results suggest that the lethality of HO-1-/- embryos occurs in late gestation stage and during birth.

**Table 1:** Genotypes of offspring from HO-1 heterozygous mice mating indicating partial embryonic lethality in HO-1 homozygous mutants

Stage	Wildtype	HO-1 (+/-)	HO-1 (-/-)	HO-1 (-/-) Expected	Total (n)
P10	68 (30.8%)	131 (59.3%)	22 (9.9%)*	55.25 (25%)	221

P1	13 (30.3%)	25 (58.1%)	5 (11.6%)	10.75(25%)	43

E18.5	10 (27.0%)	21 (56.8%)	6 (16.2%)	9.25 (25%)	37

E15.5	10 (24.4%)	22 (53.7%)	9 (21.9%)	10.25 (25%)	41

Genotypes of offspring from HO-1 heterozygous mice mating were determined by genomic PCR. Percentage of HO-1 null animals among the offspring at postnatal day 10 (P10) was significantly lower than the expected Mendelian ratio (p = 0.0001, Chi-square test). P1 animals were genotyped between 12-24 hours after birth.

### Lung alveolar defects in HO-1 knockout mice

**Figure 1:**
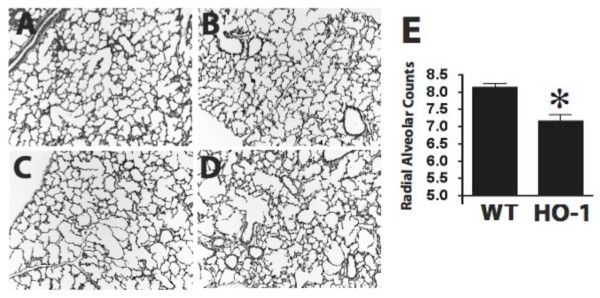
**HO-1 homozygous mutant mice display disrupted alveolar development**. A-D: H & E staining of lung sections at P10. A: Wildtype; B: HO-1 +/-; C, D: HO-1 -/-. In A and B, normal organized alveolar sac and formation of secondary septations are shown. In HO-1 homozygous mutants, alveolar development was disrupted at various severities. Panel C represents a mutant with mildly enlarged alveolar airspaces, and Panel D illustrates another mutant with more severe defects including a dramatically disorganized alveolar sac, missing septation, and a thickened interstitial region. E: Radial alveolar counts (RAC) of lung sections from wildtype (WT) and HO-1 -/- littermates at P10 (n = 4 in each group). _* _P < 0.05 *vs*. WT. The HO-1 mutants demonstrated significant decreased RAC.

Lungs from the wildtype and mutant littermates were harvested and processed for histologic and molecular analysis. At postnatal day 10, lungs from the wildtype animals had developed well-organized terminal airways consistent with alveolar sacs with secondary septations. These structures are critical to the efficient gas-exchange function of the lung (Figure 1A). The HO-1+/- lung did not reveal visible differences compared to wildtype (Figure 1B). However, the HO-1 -/- lung displayed a disorganized and simplified alveolar structure, ranging in lung defect severity (Figure 1C, D). Figure 1C represents a lung from a HO-1 -/- animal with only mild defects including slightly enlarged alveolar spaces and thinning of the alveolar wall. Figure 1D represents the lung of another HO-1 -/- animal with much more severe defects, including dramatically disorganized alveolar structure, largely missing secondary septations, enlarged alveolar spaces, and thickened interstitial regions. The HO-1 -/- animals displayed significantly decreased radial alveolar counts (RAC), a quantitative measurement of the development of the alveolar structure (Figure 1E). These data support a role for HO-1 during early postnatal alveolar formation.

### Postnatal glucocorticoid treatment caused disruption of alveolar development

**Figure 2:**
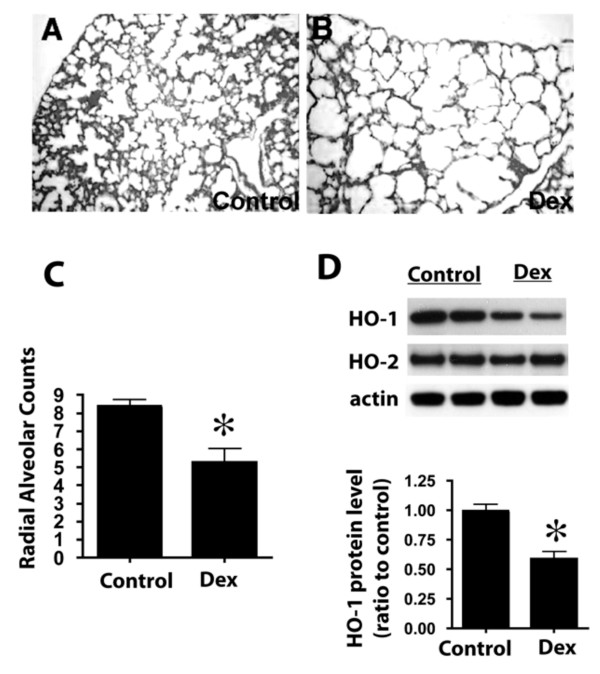
**Dexamethasone treatment disrupts postnatal alveolar development**. A, B: Representative H&E staining of mouse lung sections at P10. Wildtype newborn mice were injected daily with saline (Control in A) or 1 ug/pup dexamethasone (Dex, in B). Note the enlarged alveolar airspace and thinning of the alveolar wall in Dex-treated animals (B). C. Lung alveolar counts in control and Dex-treated mice. _* _P < 0.05 *vs*. Control. D. HO protein levels in control and Dex-injected lungs. Upper panel: representative Western blot of P10 lungs from Control and Dex-injected mice with antibodies against HO-1, HO-2 and ß-actin (loading control). Samples from two animals for each group are shown. HO-1 protein levels were visibly decreased in Dex-injected samples, whereas HO-2 protein levels remained unchanged. Lower panel: densitometric values for HO-1 protein levels normalized with ß-actin, and expressed as ratio to control. _* _P < 0.05 *vs*. Control.

Previous studies documented that postnatal corticosteroid treatment in rodent causes impaired alveolar development with inhibited secondary septation formation [39-41]. We first tested the effects of postnatal Dex treatment in newborn wildtype mice development and examined HO-1 expression in the treated lungs. Newborn wildtype C57BL6 pups were injected subcutaneously with dexamethasone from postnatal day 3 (P3) to P10 using a dose of 1 μg/pup/day, as previously published [42]. At P10, Dex-treated pups weighed approximately 10% less than the control groups, which included pups receiving no injection or saline (diluents for Dex) injections. In Dex-treated lungs, alveolar walls were thin, secondary septations were incomplete, distal airspaces were significantly larger and simplified, resulting in loss of alveolar surface area for gas exchange (Figure 2 A, B). The RAC measurements in the Dex group were significantly decreased compared to controls (Figure 2C), indicating enlarged alveoli. Intriguingly, HO-1 protein level decreased by approximately 45% in the lungs from the Dex-treatment animals at P10. Protein levels of the non-inducible HO-2 isoform did not change after Dex injection (Figure 2D). These results demonstrate that Dex significantly inhibits postnatal alveolar formation and that HO-1 expression is dramatically repressed by Dex treatment, suggesting that negative regulation of HO-1 protein by Dex might contribute to the alveolar defects observed.

### Dex treatment in HO-1 knockout animals exacerbated lung defects

**Figure 3:**
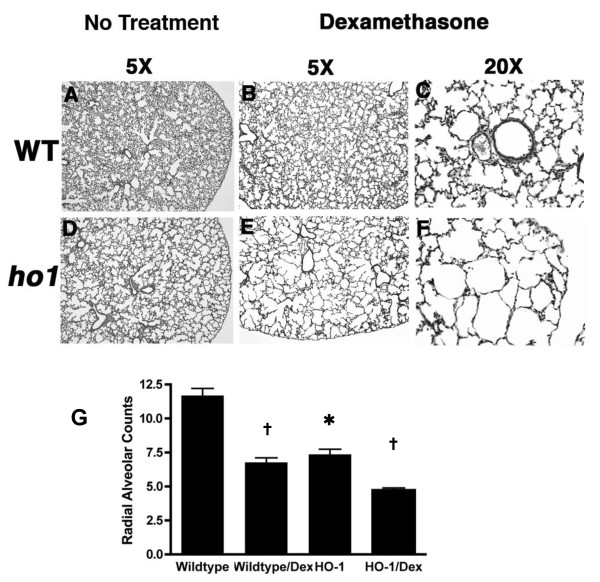
**Dexamethasone treatment exacerbates the alveolar defects in HO-1 mutant mice**. A-F: Representative H&E staining of mouse lung sections at P14 at 5X and 20X magnifications. A-C: Wildtype; D-F: HO-1 -/-. A, D: lungs from untreated animals. B, C, E, F: lungs from Dex-treated animals (P3-P14, 0.25 ug/pup/day). In untreated group, lungs from HO-1-/- animals showed simplified, enlarged, and disorganized alveolar structure (A, D). Postnatal Dex treatment in wildtype animals resulted in alveolar simplification and loss of secondary septation (A, and B, C). Dex treatment in HO-1 -/- animals resulted in more dramatic disruption of the alveolar structure with larger alveolar space, thinning of the alveolar wall, and lack of secondary septation. V: pulmonary vasculature. A: airway. Arrowhead indicates the normal secondary septae. Arrows indicate the elongated and thinning of the alveolar wall. G: Quantification of alveolar development by RAC of the lung sections at P14. _* _P < 0.05 *vs*. wildtype, † P < 0.05 *vs*. untreated group of same genotype. n = 3-4 for each group.

We further evaluated the effect of postnatal Dex treatment on HO-1 -/- newborn mice. In our previous experiment with wildtype C57BL6 mice (Figure 2), a Dex dose of 1 μg/pup was used. This was based on published studies [42] as well as work in our own lab. However, none of the HO-1 mutant animals survived the 1 μg/pup dose. We therefore reduced the Dex dose to 0.25 μg/pup and treated the entire litter from P3 to P14. This protocol resulted in no difference of survival rate in the treated and control HO-1 mutants. Consistent with our data at earlier time point P10, HO-1 mutant lungs at P14 displayed defective alveolar structure at baseline, without Dex treatment. The phenotypes include bigger alveolar space, reduced complexity, and reduced secondary septation formation (Figure 3D vs. 3A). RAC measurement demonstrated a significantly reduced alveolar number in HO-1 mutant lung (RAC = 7.4), 37% less of the wildtype value (RAC = 11.7) (Figure 3G). After Dex treatment, the HO-1 mutant lung had much worse disruption of alveolarization than that of the wildtype littermates (Figure 3B-F). Dex treatment in wildtype animals resulted in inhibition of alveolar formation. With the reduced dose of Dex, the effect (Figure 3B, C) is milder than the effect shown in Figure 2. However, in the Dex-treated HO-1 -/- lung, the alveolar space was dramatically enlarged; the alveolar lining was thinning, and the overall alveolar architecture was simplified. Most strikingly, the formation of secondary septation in the alveoli, an event essential in generating a sufficient gas exchange area, was largely abolished in the mutant lung, suggesting a significant loss of gas exchange surface and compromised pulmonary function (Figure 3E, F). Quantification of alveolar formation by RAC displayed that the Dex treatment further lowered the alveolar count to 4.7 in the HO-1 mutants, a 60% decrease from the wildtype no treatment group (Figure 3G). To determine if HO-1 deficiency and Dex treatment change the regulation of cell death, we performed TUNEL assay on P14 lung sections. There was no significant difference between the different genotypes and different treatment groups (data not shown).

### Down-regulation of lung epithelial and vascular genes in Dex-treated HO-1 mutants

**Figure 4:**
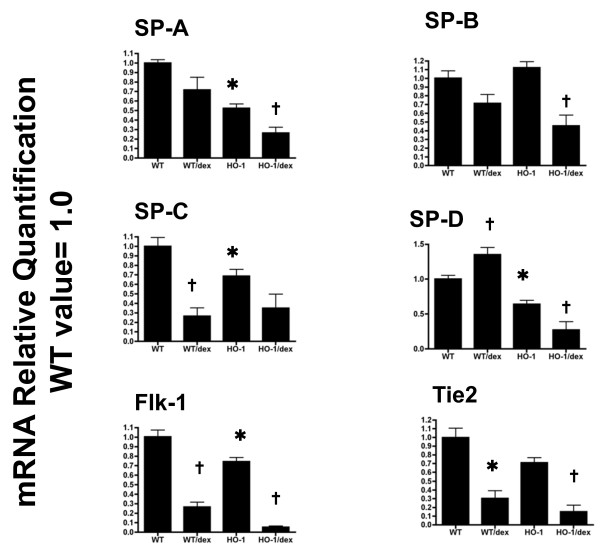
**Expression of lung epithelial and vascular genes in wildtype and HO-1 -/- after dexamethasone treatment**. Gene expression of surfactant protein (SP)-A, -B, -C, and D, as well as Flk-1 and Tie-2 was determined by realtime PCR and normalized to 18 S mRNA levels. Data represents relative quantification of mRNA to wildtype littermates at baseline (no treatment). _* _P < 0.05 *vs*. WT, † P < 0.05 *vs*. untreated group of same genotype. n = 3-4 for each group.

To evaluate the maturation of the lung alveolar cells, we further examined the mRNA levels of pulmonary type II epithelial cell markers in these animals. Surfactant Proteins (SP) genes are a family of genes specific for type II cells and essential for type II cell function. We assessed all four surfactant protein genes by quantitative real-time PCR with gene specific probes and primers. At baseline of P14, expression levels of three surfactant proteins, -A, -C, and -D, were significantly lower in HO-1 mutant lungs. No difference in SP-B expression was detected between HO-1 mutant and WT littermates. Dex treatment in wildtype resulted in decreased levels of SP-A, -B, and -C, and an increase in SP-D. However, after Dex treatments in the HO-1 null mutant, all surfactant gene mRNAs were decreased compared to the untreated group (Figure 4A-D).

It is well reported that pulmonary vascular development is critical to postnatal alveolar formation. Previously published results also demonstrated that the VEGF receptor-2 (KDR/Flk-1) was down-regulated in Dex-treated neonatal mice with reduced alveolarization [40]. We further examine the expression of two endothelial cell markers, Flk-1 and Tie-2, in the different animal groups. At baseline, Flk-1 and Tie-2 expression in the HO-1 mutant have 26% and 29% decrease compared to wildtype littermates. Consistent with the published report, Flk-1 and Tie-2 expression both decreased significantly after Dex treatment in the wildtype, 74% and 70% respectively, from the values of the untreated group. Although it is well known that Dex has inhibitory effects on alveolar formation, these data demonstrate that pulmonary angiogenesis is also significantly inhibited by Dex via down-regulation of VEGF and Ang-mediated pathways. Most interestingly, the Dex-mediated decrease of mRNA expression levels of endothelial cell markers, including both Flk-1 and Tie-2, further decreased in HO-1 mutant after Dex treatment. The expression levels of Flk-1 and Tie-2 in the Dex treated HO-1 mutant group decreased to 7% and 15% of the values observed in the wildtype untreated group (Figure 4).

In summary, these data demonstrated that HO-1 is critical to postnatal alveolar development and that it is involved in epithelial cell growth regulation. The effects of HO-1 disruption are also additive to those of postnatal corticosteroid exposure.

## Discussion

In this paper we show with histology and molecular analysis that postnatal lung development is altered in HO-1 knockout mice. We also document that Dex treatment exacerbates the alveolar defects seen with HO-1 disruption.

HO-1 null mice display partial penetrance of embryonic lethality. Although the exact cause and the underlining mechanism are not yet determined, our preliminary data (Q. Lin, unpublished) suggest that defects in the embryonic vasculature might significantly contribute to the early lethality. In the present manuscript, we studied lung development in the HO-1 -/- mutants that survive to the postnatal period. Compared to the wildtype littermates, HO-1 -/- mutants display defects in lung alveolar development with a range of severity, including disorganized alveolar structure, thickening of the interstitial section, and loss of air exchange surface area. These loss-of-function phenotypes indicate that HO-1 is essential to normal postnatal lung development *in vivo*. Anti-oxidant enzymes have been shown to protect the lung from oxidative injury. For example, in Type II epithelial cells from newborn mice, over-expression of extracellular superoxide dismutase (EC-SOD) preserved type II cell proliferation and protected the lung from hyperoxic injury [43]. Mice deficient in endothelial nitric oxide synthase (e-NOS) displayed defective lung vascular development, which resembles the alveolar capillary dysplasia in infants with Persistent Pulmonary Hypertension of the Newborn (PPHN). It is intriguing that the antioxidant HO-1 is not only protective against stress and injury, but is also required for normal embryonic development and postnatal alveolar formation. The exact mechanism by which this occurs is not yet elucidated.

The HO-1 null mice display its phenotypic defects with a range of severity, from the viability rate to the lung and vascular disruptions. In our data analysis, this partial penetrance is the cause of more variation among the KO samples compared to the WT samples. This sometimes resulted in not achieving the statistical significance threshold of p < 0.05, even with large differences in mean values. Partial or incomplete penetrance of phenotypes is not rare and the exact molecular mechanism for this is unknown. However, this may indicate that HO-1 protein plays an important role in maintenance of the delicate homeostasis of many cellular events.

Postnatal steroid therapy benefits preterm infants by accelerating lung maturation, reducing lung inflammation and facilitating extubation from the ventilator. However, the adverse effects associated with glucocorticoid usage are significant and there also may be detrimental long-term damage to the brain and lung. In the lung, dexamethasone can impair lung septation and alveolar formation in the early postnatal period. Furthermore, the interrupted alveolar development does not resume normally even after treatment stops. In our experiment, we have shown that Dex treatment in the postnatal period cause significant loss of alveolar complexity and decreased HO-1 protein levels in the wildtype lung. This reduction of the cytoprotective molecule HO-1 may contribute to the abnormal alveolar growth in the treated animals. Previous studies have also reported that HO-1 expression can be suppressed by Dex in cultured endothelial cells [44,45]. Genomic analysis of the HO-1 gene promoter and enhancer regions reveals at least four putative glucocorticoid receptor (GR) binding sites, indicating transcriptional repression via direct binding of GR to the HO-1 gene regulatory regions. Nonetheless, repression can be achieved through many mechanisms including epigenetic regulation or chromatin remodeling, posttranslational modifications, and protein-protein interactions. However, this was not specifically tested in the current work.

HO-1 mutant mice have reduced viability during embryonic development and postnatally. The cause of the postnatal lethality is not fully understood yet. In this paper, we reported lung structural defects in HO-1 mutant animals including enlarged alveolar spaces, and simplified alveolar structure with less secondary septae. These defects would result in reduced gas exchange surface area of the lung and lead to compromised pulmonary function, hence the postnatal mortality.

When we subjected the HO-1 mutant to Dex treatment, the dosage previously used on wildtype animal at the same age was lethal to HO-1 mutants. We then reduced the Dex dose to 25% of the original. This resulted in milder alveolar simplification in the WT, but dramatic defects in the HO-1 mutants with decreased radial alveolar counts, thinner alveolar wall, and inhibited secondary septae formation. This suggested that the effects of HO-1 disruption and dexamethasone treatment are additive. Since mice lacking HO-1 show further disrupted lung development when treated with steroids, the data suggest that HO-1 and steroid-mediated disruption of lung development are independent but additive.

Gene array experiments identified that critical vascular genes, including Flk-1, were down-regulated in the Dex-treated animals, suggesting that pulmonary vascularization in the developing lung is critical to postnatal alveolarization [40]. We have observed a similar decrease in Flk-1 after Dex treatment in the current study. In addition, we have examined another endothelial marker Tie-2, and confirmed that Dex treatment caused significant decrease in endothelial gene expression. In BPD and in other neonatal lung diseases with arrested and impaired alveolar development, defects in pulmonary vasculature, such as decreased blood vessel density, abnormal vessel branching patterns and down-regulation of vascular growth factors, have also been identified. Furthermore, studies also found that in addition to decreased endothelial content, proliferation, migration and survival of these cells may also be compromised in BPD [46]. Altogether, increasing evidence suggests that the proliferation, differentiation, and patterning of vascular endothelial and epithelial lineages in the lung may exert a reciprocal influence on lung morphogenesis and growth.

Previous studies demonstrated that HO-1 is involved in vascular development by facilitating blood vessel formation in tumors, wounds, and experimental models of angiogenesis [19,29]. In cultured endothelial cells, induction of HO-1 and increased CO levels up-regulate the expression of VEGF and VEGF receptors, increase endothelial cell proliferation, migration and sprouting, and promote angiogenesis [47-49]. HO-1 induction or CO exposure in vascular smooth muscle cells also up-regulates the expression of VEGF [50]. These data indicate that induced HO-1 may function in the vascular system by counteracting the deleterious effects of reactive oxygen species (ROS) and by producing CO as a vasorelaxant and regulator of vascular growth. In our current study, we have shown that Dex-treated HO-1 knockout mice have dramatically disrupted alveolar development. Interestingly, vascular gene expression was even more significantly decreased in HO-1 mutant mice after Dex treatment. In addition, severe vascular defects are found in HO-1 mutant embryos (unpublished data). Thus, we speculate that the exacerbated lung alveolar defects observed after Dex treatment in HO-1 null mutant mice might result from the disrupted pulmonary vasculature.

Postnatal glucocorticoid usage in preterm infants can facilitate lung maturation and reduce lung inflammation, yet it can have detrimental effects on lung and neural development. During postnatal lung growth, Dex treatment causes loss of alveolar septation, which results in a large, simplified alveolar structure with decreased gas exchange surface area. In addition, increased oxidative stress contributes to neonatal lung disease by affecting alveolar growth. Our findings, that Dex treatment decreases HO-1 expression and that disruption of HO-1 protein results in more severe vascular and alveolar defects after Dex treatment, suggest that enhancing this important antioxidant system might be a beneficial strategy to obviate neonatal lung disease.

There are several limitations of the current study. Firstly, the sample sizes of HO-1 mutant animals in each experiment group in the study were small. This is due to the difficulty of obtaining viable HO-1 null newborns. With the partial penetrance of the phenotype, in some assays, the data variation is bigger than the WT control group. With a bigger breeding colony or in vitro fertilization techniques using gametes from homozygous and heterozygous animals, we will be able to generate more HO-1 mutant animals for future studies. Secondly, we have demonstrated structural defects as well as gene expression alterations, but no functional assessment was conducted. In future studies, we can measure pulmonary function in the animals as a correlate. Thirdly, further genetic analysis with more tools is needed to establish the molecular mechanism connecting glucocorticoids to HO-1. For example, it would be useful to evaluate the effect of Dex in the HO-1 transgenic over-expressors.

## Conclusions

In summary, we show evidence that HO-1 deficiency in the mouse results in disrupted postnatal alveolar development including abnormal alveolar structure and decreased epithelial and endothelial marker expression. These defects were further exacerbated when the HO-1 mutant animals were treated with glucocorticoids. The decrease in endothelial gene expression was more dramatic than that of the lung epithelial markers. These experiments demonstrate that HO-1 is required for normal lung development and that HO-1 disruption and dexamethasone have additive detrimental effects on postnatal lung growth. We speculate that HO-1 is involved in postnatal lung development through modulation of pulmonary vascular development.

## Competing interests

The authors declare that they have no competing interests.

## Authors' contributions

TZ performed the molecular biology experiments in the manuscript and participated in its design. MZ performed part of the animal studies and radial alveolar counts. HZ supervised lung morphology analysis and assisted in data analysis. PAD participated in study design, data interpretation and manuscript editing. QSL conceived of the study, participated in its design and execution, performed animal studies, and wrote the manuscript. All authors read and approved the final manuscript.
